# Integrating Network Pharmacology, Machine Learning, and Experimental Validation to Elucidate the Mechanism of Cardamonin in Treating Idiopathic Pulmonary Fibrosis

**DOI:** 10.3390/ijms27010249

**Published:** 2025-12-25

**Authors:** Wenyue Zhang, Yi Guo, Qiushi Wang, Kai Wang, Huning Zhang, Sirong Chang, Anning Yang, Zhihong Liu, Yue Sun

**Affiliations:** 1 School of Public Health, Ningxia Medical University, Yinchuan 750000, China; 2 Key Laboratory of Metabolic Cardiovascular Disease Research, National Health Commission, Ningxia Medical University, Yinchuan 750000, China

**Keywords:** Cardamonin, network pharmacology, machine learning, idiopathic pulmonary fibrosis

## Abstract

Idiopathic pulmonary fibrosis (IPF) is a chronic and irreversible interstitial lung disease characterized by progressive scarring of the lungs. The available therapeutic strategies are limited and primarily focus on slowing disease progression rather than achieving fibrosis reversal. Cardamonin (CDN), a food-derived natural chalcone, has exhibited anti-fibrotic activity in liver and kidney fibrosis models; however, its role and underlying mechanism in IPF remain unelucidated. Herein, we integrated network pharmacology, machine learning, molecular simulations, and in vitro experiments. Network pharmacology identified 135 overlapping targets between CDN and IPF, which demonstrated a significant enrichment in the Phosphatidylinositol 3-Kinase/Protein Kinase B signaling pathway (PI3K/AKT). Machine learning further prioritized 6 core targets, with IGF1 emerging as a key candidate. Molecular docking revealed a favorable binding energy of −7.9 kcal/mol for the CDN-IGF1 complex. Subsequent 100 ns molecular dynamics simulations further confirmed its robust binding stability, yielding a mean binding free energy of −150.978 kcal/mol. In vitro, CDN significantly mitigated fibrosis in bleomycin (BLM)-challenged A549 cells, downregulating the expression of α-smooth muscle actin (α-SMA) and fibronectin. This effect was accompanied by a beneficial reversal of epithelial–mesenchymal transition (EMT), as indicated by increased E-cadherin levels and decreased vimentin expression. Mechanistically, CDN significantly suppressed the IGF1/PI3K/AKT axis; this inhibitory effect was partially reversed by exogenous IGF1 supplementation and further enhanced by the PI3K-specific inhibitor LY294002. This work provides the evidence that CDN alleviates BLM-induced pulmonary fibrosis by targeting the IGF1/PI3K/AKT-EMT axis. These findings lend support to a robust mechanistic basis for developing CDN as a potential therapeutic candidate for IPF. It should be noted that these conclusions are drawn from in vitro experiments using A549 cells, and further validation in primary alveolar epithelial cells and animal models is warranted to confirm their physiological relevance.

## 1. Introduction

Idiopathic Pulmonary Fibrosis (IPF) is a lethal interstitial lung disease driven by a dysregulated repair process. This condition features aberrant extracellular matrix (ECM) accumulation and progressive destruction of the alveolar architecture, culminating in a relentless decline in pulmonary function [1]. As its incidence increases with age and it has a median post-diagnosis survival of merely 2 to 5 years, IPF poses a significant threat to global respiratory health [2]. Currently available first-line drugs pirfenidone and nintedanib merely delay disease progression and cannot reverse established fibrosis. Moreover, these drugs are often associated with adverse effects such as nausea, diarrhea, and fatigue, leading to insufficient patient compliance [3,4]. Moreover, IPF’s complex pathogenesis, involving epithelial–mesenchymal transition (EMT), persistent inflammation, oxidative stress, and dysregulated signaling pathways, remains incompletely understood. This mechanistic gap hinders the development of targeted therapies, creating an urgent need for innovative strategies to identify potential therapeutic agents and decipher their anti-fibrotic mechanisms.

As a natural chalcone compound, Cardamonin (CDN) is mainly extracted from plants belonging to the Zingiberaceae family, such as Alpinia katsumadai Hayata [5]. These traditional Chinese medicinal materials have long been used for regulating gastrointestinal function and reducing inflammation. Extensive pharmacological profiling of CDN has revealed a spectrum of beneficial activities, encompassing anti-inflammatory, antioxidant, and anti-tumor [6,7]. Specifically, CDN has been shown to normalize glycolytic flux in hepatocytes by suppressing mammalian target of rapamycin (mTOR) activation [8,9]. Preclinical evidence further indicates that CDN ameliorates fibrosis in both the liver and kidneys. The underlying mechanism involves the consistent suppression of pro-fibrotic pathways, such as Transforming growth factor-beta (TGF-β)/Smad and Nuclear factor kappa-light-chain-enhancer of activated B cells (NF-κB) signaling [10,11]. However, there are currently no studies that systematically evaluate whether CDN can alleviate pulmonary fibrosis, and its potential molecular targets, key signaling pathways, and anti-IPF mechanisms remain unclear. This research gap makes CDN a promising candidate for the development of novel IPF therapeutic drugs, while also highlighting the necessity of conducting in-depth mechanistic studies on it.

Considering the complex crosstalk among signaling pathways in the IPF pathological network and inherent multi-targeted property of natural products such as CDN, traditional single-target research methods are no longer sufficient to fully reveal their therapeutic mechanisms. We employ two powerful tools—network pharmacology and machine learning. Among these, network pharmacology can systematically predict the potential molecular targets of natural products and identify overlapping pathogenic genes in diseases, thereby overcoming the limitations of traditional single-target research [12]. Meanwhile, machine learning (e.g., K-nearest neighbor (KNN) algorithm, Least Absolute Shrinkage and Selection Operator (LASSO) regression, and random forests) effectively reduce the false positive rate and focus on pathways most relevant to disease progression by prioritizing core mediator molecules based on importance scores [13]. This study adopts an integrated framework of “computational prediction–experimental validation” to systematically elucidate the anti-IPF mechanism of action of CDN, which mainly consists of three steps: First, it utilizes network pharmacology to screen the potential human targets of CDN, intersect these targets with IPF-related targets, construct a core interaction network, and identify candidate pathways. Second, it applies machine learning (e.g., LASSO regression, artificial neural networks) to integrate public IPF omics datasets, prioritize core targets with high importance, and optimize mechanistic hypotheses. Finally, it conducts in vitro experiments to validate the predicted targets and pathways, clarifying the regulatory effect of CDN on cell proliferation, ECM deposition, and EMT.

The present study aims to verify the therapeutic efficacy of CDN in IPF and explore its specific anti-fibrotic mechanism of action. By doing so, we seek to provide a theoretical basis for developing CDN as a natural, therapeutic candidate for this refractory disease.

## 2. Results

### 2.1. In Vitro Antifibrotic Effects of CDN

**Figure 1:**
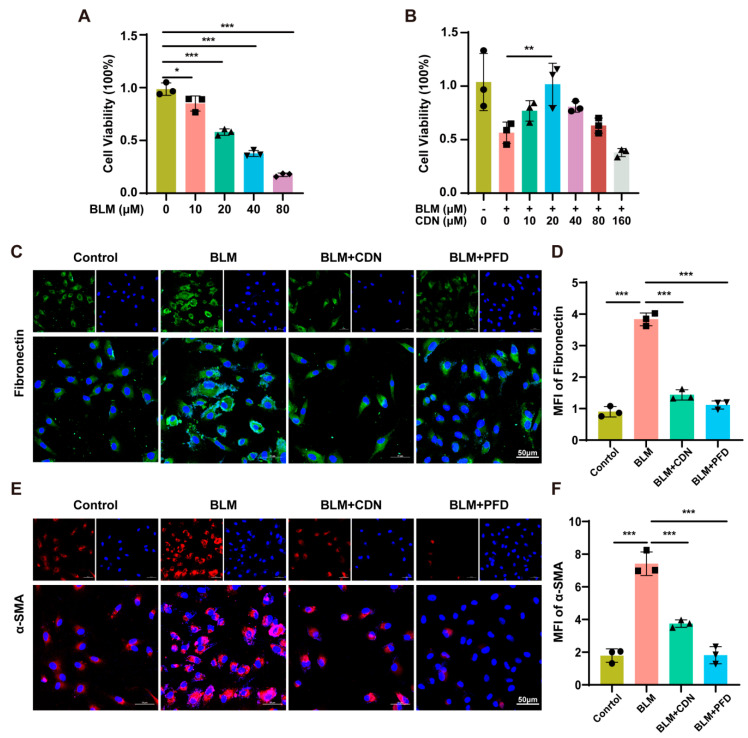
Cardamonin (CDN) mitigates bleomycin (BLM)-induced fibrotic responses in A549. (**A**) Dose-dependent reduction of A549 viability by BLM (0–80 μM). (**B**) CDN concentration-dependent effects on BLM-induced cytotoxicity. (**C**) Immunofluorescence staining and (**D**) Fibronectin expression quantification. (**E**) α-smooth muscle actin (α-SMA) immunofluorescence staining (red) and (**F**) α-SMA expression quantification. Nuclei: blue (DAPI); Fibronectin: green; α-SMA: red. Scale bars: 50 μm. Data are presented as mean ± SD (*n* = 3); * *p* < 0.05, ** *p* < 0.01, *** *p* < 0.001.

Initial dose optimization via Cell Counting Kit-8 (CCK-8) assay established appropriate concentrations of bleomycin (BLM) and CDN. Exposure to 20 µM BLM reduced A549 cell viability while maintaining sufficient cellular integrity for phenotypic assessment (Figure 1A). CDN administration concentration-dependently restored viability in BLM-injured A549 cells, with maximal cytoprotective efficacy observed at 20 µM (Figure 1B). Further analysis of CCK-8 results confirmed two key findings: (1) CDN concentrations up to 80 µM did not significantly reduce the viability of untreated A549 cells (vs. 0 µM CDN group, *p* > 0.05), identifying 80 µM as the maximum non-toxic concentration (MTC) of CDN; (2) Among the tested concentrations, 20 µM CDN exerted the most potent cytoprotective effect—effectively restoring the viability of BLM-injured A549 cells to ~85% of the control level (vs. BLM-only group, *p* < 0.001)—whereas higher concentrations (40, 80 µM) failed to further enhance this protective effect (*p* > 0.05 vs. 20 µM CDN group). Additionally, subsequent immunofluorescence experiments verified that 20 µM CDN most significantly suppressed BLM-induced upregulation of fibrotic markers (α-SMA, fibronectin) (Figure 1C–F). Together, these data demonstrate that 20 µM CDN balances both safety (non-toxicity to normal A549 cells) and efficacy (potent cytoprotection and anti-fibrotic activity), thus being selected as the primary concentration for all subsequent in vitro experiments. Collectively, these findings indicate that CDN effectively attenuates BLM-induced fibrotic responses in human alveolar epithelial cells in vitro.

### 2.2. Identification of Potential Targets of CDN

To comprehensively explore the antifibrotic mechanisms of CDN, we employed a network pharmacology approach by interrogating multiple public databases with distinct prediction algorithms, including Swiss Target Prediction (based on 2D/3D structural similarity), Encyclopedia of Traditional Chinese Medicine (ETCM) (herbal medicine-specific), PharmMapper (pharmacophore mapping), Search Tool for Interacting Chemicals (STITCH) (chemical-protein interactions), and SuperPred (similarity-based and machine learning). The canonical SMILES of CDN was used as the query input for all databases, and a probability cutoff of >0.7 was applied to retain only high-confidence predictions. This initial screening yielded 100, 23, 304, 6, and 74 potential targets from the respective databases. The combined list was then standardized by mapping all target names to official gene symbols using the UniProt database. After removing duplicates, a non-redundant set of 441 unique potential targets of CDN was established for subsequent analysis (Table S1, Supporting Information).

### 2.3. Target Screening for IPF

**Figure 2:**
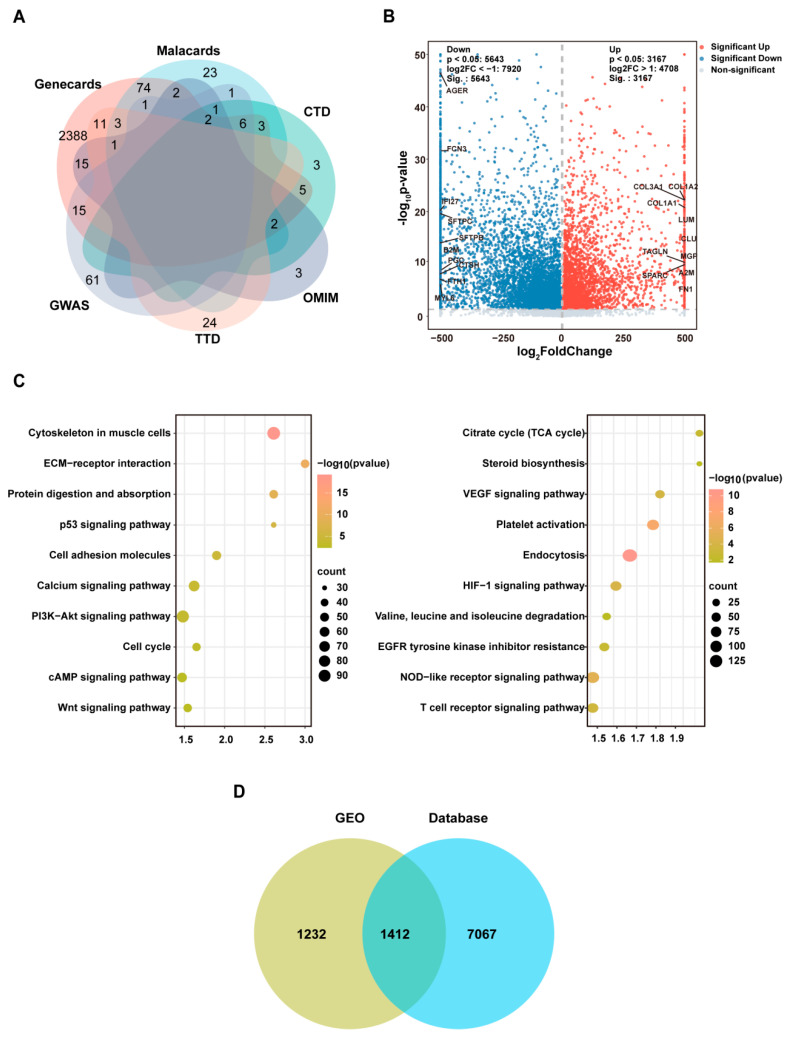
Discovery of candidate genes for Idiopathic Pulmonary Fibrosis (IPF). (**A**) Venn diagram of IPF-associated targets from Genecards, Malacards, Comparative Toxicogenomics Database (CTD), Online Mendelian Inheritance in Man (OMIM), Therapeutic Target Database (TTD), and Genome-Wide Association Study (GWAS) databases. (**B**) Volcano plot of differentially expressed genes (DEGs) in IPF (threshold: |log2(fold change)| > 1). The horizontal dashed line indicates the threshold for statistical significance (*p* = 0.05). The two vertical dashed lines represent the thresholds for a 2-fold change (log_2_ FoldChange = ±1). (**C**) Kyoto Encyclopedia of Genes and Genomes (KEGG) pathway enrichment analysis of up/downregulated DEGs. (**D**) Intersection of IPF targets from gene cards, mala cards, CTD, OMIM, TTD, and GWAS and the Gene Expression Omnibus (GEO) public gene expression dataset (GEO accession: GSE24206, GSE150910, GSE213001, and GSE124685).

This study integrated data from five public databases and Gene Expression Omnibus (GEO) datasets: specifically, a total of 2528 protein-coding genes were retrieved from the GeneCards database, 26 genes from the Online Mendelian Inheritance in Man (OMIM) database, 26 genes were screened from 341 associated genes in the MalaCards database, 22 genes from the Comparative Toxicogenomics Database (CTD), and 87 genes from the Genome-Wide Association Study (GWAS) Catalog. After integration of the aforementioned data, a high-confidence gene list containing 2661 genes was generated (Figure 2A).

After extracting data from four datasets (GSE24206, GSE150910, GSE213001, and GSE124685) in the GEO database, we first employed the ComBat method for batch correction. Subsequently, we verified the consistency of sample distribution via principal component analysis (Figure S1A). Next, inter-group differential expression analysis was conducted using the Limma package to identify significant genes, applying thresholds of |logFC| > 1 and FDR-adjusted *p*-value < 0.05. Our analysis identified 8479 genes as differentially expressed (DEGs). These were further categorized into 3167 upregulated and 5643 downregulated genes (Figure 2B). The most significant enrichments from the Kyoto Encyclopedia of Genes and Genomes (KEGG) analysis of DEGs are displayed in Figure 2C. Upregulated genes were strongly associated with the p53 and PI3K-Akt signaling pathways, whereas the Nucleotide-binding oligomerization domain (NOD)-like receptor, Hypoxia-inducible factor 1 (HIF-1), and Vascular endothelial growth factor (VEGF) signaling pathways were prominently enriched for downregulated genes.

Finally, an intersection analysis was conducted to integrate the fibrosis-related genes from public databases with the DEGs sourced from the GEO database. This integrative approach ultimately identified 1412 key genes implicated in IPF (Figure 2D).

### 2.4. Enrichment Analysis and Protein–Protein Interaction (PPI) of Important Targets

**Figure 3:**
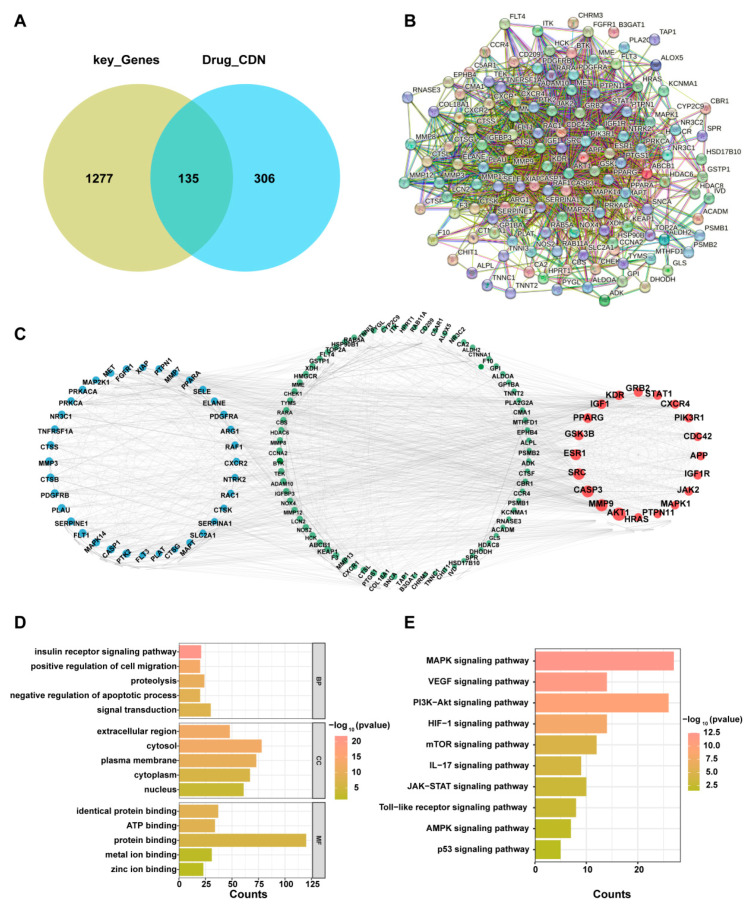
Analysis of Gene Interactions and Pathway Enrichment. (**A**) Venn diagram depicting the overlap between key genes of IPF and CDN. (**B**,**C**) Protein–protein interaction (PPI) network among key genes. (**D**) Bar graph of enriched Gene Ontology (GO) biological processes. (**E**) Bar graph of enriched KEGG pathways.

According to the Venn diagram, CDN has 135 potential targets in IPF (Figure 3A). A PPI network was constructed for these genes using the Search Tool for the Retrieval of Interacting Genes/Proteins (STRING) database, yielding a network of 135 nodes and 1402 edges (Figure 3B,C). Gene Ontology (GO) analysis revealed significant enrichment in the biological process (BP) category for terms including positive regulation of cell migration, negative regulation of the apoptotic process, and signal transduction. Cellular components (CC) were enriched in extracellular region and cytosol. Molecular Function (MF) analysis highlighted prominent enrichment for roles in identical protein, ATP binding, and general protein binding (Figure 3D). Additionally, KEGG pathway analysis implicated a set of key signaling pathways, most notably MAPK, VEGF, and PI3K-Akt, in the functional profile of these genes (Figure 3E).

### 2.5. Machine Learning and Molecular Docking Screening of Core Targets of CDN in IPF Treatment

**Figure 4:**
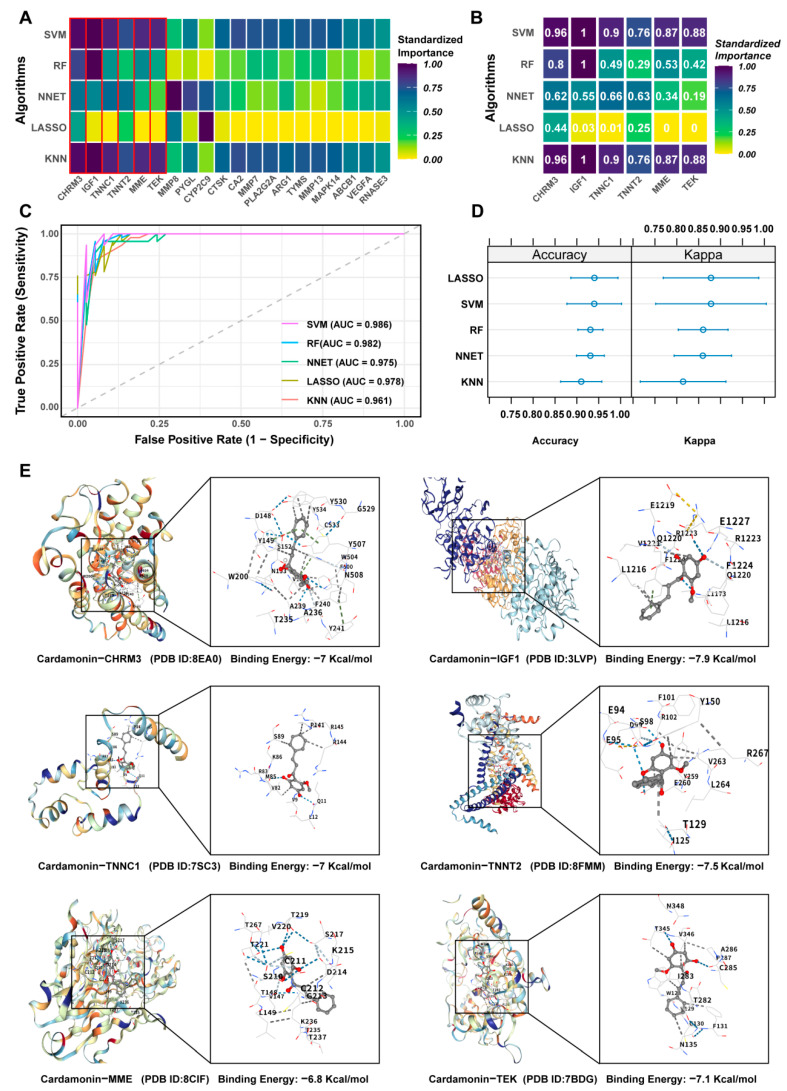
Feature Importance and Molecular Docking Analysis. (**A**) Feature Importance Heatmap Across Machine Learning Algorithms for the Top 20 Genes Ranked by Average Importance. The red box highlights the Top 6 feature genes selected by the machine learning models. (**B**) Heatmap of Core Genes’ Importance Across Machine Learning Algorithms. (**C**) Receiver Operating Characteristic (ROC) curves of various models (with Area Under the Curve (AUC) values). The diagonal dashed grey line represents the performance of a random classifier (AUC = 0.5). (**D**) Dot plots of accuracy and Kappa coefficients. (**E**) Molecular docking results.

To identify the core targets of CDN in IPF, we trained five machine learning algorithms (KNN, LASSO, Artificial Neural Network (NNET), Random Forest (RF), and Support Vector Machine (SVM)) and ensured model robustness through 5-fold cross-validation combined with preprocessing steps (centering and standardization). Through feature importance analysis, we presented a heatmap of the top 20 genes ranked by average importance across all models (Figure 4A). Core genes such as CHRM3, IGF1, TNNC1, MME, and TEK maintained stably high importance across different algorithms (Figure 4B), confirming their characteristic of being prioritized by multiple models. Model performance was evaluated using Receiver Operating Characteristic (ROC) curves (Figure 4C): the Area Under the Curve (AUC) values of all five machine learning algorithms (KNN, LASSO, NNET, RF, and SVM) exceeded 0.95, all indicating strong discriminative ability for IPF-related features. The dot plots of accuracy and Kappa coefficients (Figure 4D) further verified the stability and consistency of performance across models, with a narrow distribution range of metrics, demonstrating the reliability of the results.

On the basis of identifying core targets and verifying the reliability of their prediction through machine learning, we further conducted molecular docking analysis to explore the interaction characteristics between CDN and these core target proteins. Molecular docking (Figure 4E) showed that the binding energies of CDN with core target proteins (CHRM3, IGF1, TNNC1, TNNT2, MME, TEK) ranged from −6.8 to −7.9 kcal/mol (more negative values indicate stronger affinity), confirming that CDN forms stable interactions with these proteins. In summary, core targets with high predictive value were screened out by machine learning algorithms, and molecular docking verified the interaction ability between CDN and these targets, laying a foundation for in-depth exploration of the therapeutic mechanism of CDN in IPF.

### 2.6. Molecular Dynamics Analysis

**Figure 5:**
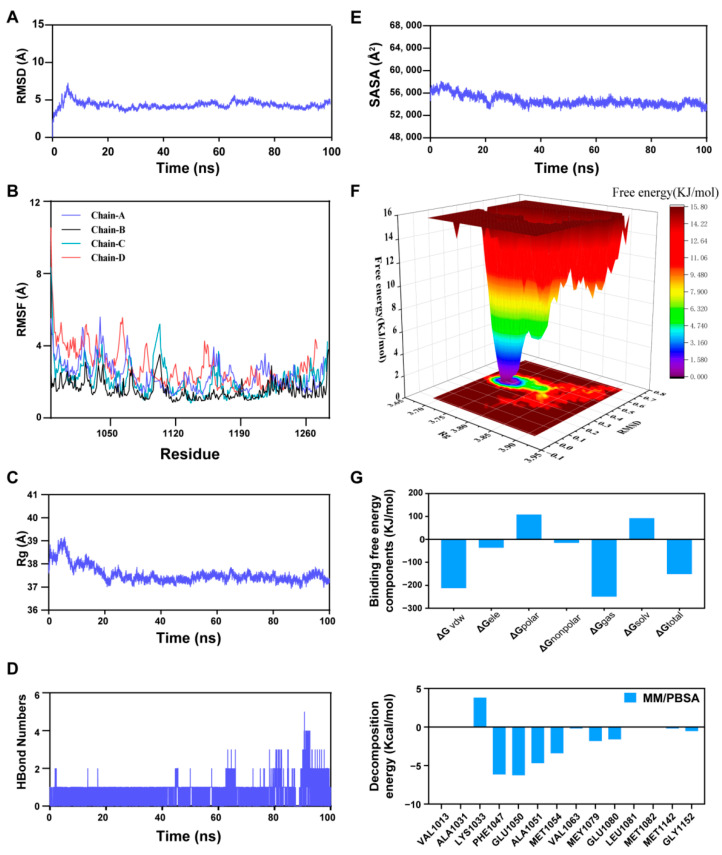
Molecular Dynamics Simulation Analysis of the IGF1-CDN Complex. (**A**–**C**) Structural stability assessed by Root Mean Square Deviation (RMSD) (**A**), Root Mean Square Fluctuation (RMSF) (**B**), and Radius of Gyration (Rg) (**C**). (**D**,**E**) Interaction properties characterized by hydrogen bond count (**D**) and Solvent Accessible Surface Area (SASA) (**E**). (**F**) Free Energy Landscape (FEL) of the IGF1-CDN Complex. (**G**) Decomposition of Binding Free Energy Components and Residue Energy Contribution Analysis.

To further investigate the dynamic stability and key interaction details of the interaction between CDN and core targets, based on a comprehensive evaluation of the results from previous machine learning algorithms and molecular docking, we prioritized selecting IGF1—with the lowest binding energy (−7.9 kcal/mol)—as the representative target to conduct molecular dynamics (MD) simulation analysis. The structural stability of the protein–ligand complex throughout the simulation was evaluated by monitoring the Root Mean Square Deviation (RMSD) [14,15]. Reduced RMSD values indicate limited conformational alterations and improved structural stability of the complex. Root Mean Square Fluctuation (RMSF) curves, by contrast, demonstrate the fluctuation amplitude of amino acid residues throughout molecular dynamic simulations. A direct correlation exists between RMSF values and residue mobility: elevated readings correspond to enhanced flexibility, whereas diminished values reflect more constrained atomic movements [16,17]. We utilized the Radius of Gyration (Rg) to quantify the overall structural tightness. Higher Rg values correspond to a more extended or loosened architecture, while lower values reflect a stable, condensed conformation [18]. For the IGF1-CDN complex system, equilibrium was attained at 70 ns, with RMSF values maintaining a comparatively low level (fluctuating around 5 Å). These experimental findings demonstrate that the incorporation of CDN exerted negligible effects on the stability of amino acid residues within IGF1, and the formed complex manifested desirable stability (Figure 5A–C). Furthermore, the quantity of hydrogen bonds formed between IGF1 and CDN stayed steady, exhibiting only slight fluctuations. Solvent Accessible Surface Area (SASA) is a core parameter for measuring the size of the regions on small molecules (ligands) and proteins that are exposed to solvents [19]. Our findings revealed that the protein–small molecule complex system presented slight variations, which indicated that the binding of small molecules modified the local binding microenvironment and brought about partial changes in SASA (Figure 5D,E). The Free Energy Landscape (FEL) also revealed the desirable stability of the IGF1-CDN complex (Figure 5F). Subsequent to the stability confirmation, the Molecular Mechanics/Poisson–Boltzmann Surface Area (MM/PBSA) approach was used for the determination of the average binding free energy in the IGF1-CDN complex. The average binding free energy of the IGF1-CDN complex, as calculated, was determined to be −150.978 kcal/mol, which points to a potent binding affinity between the IGF1 protein and CDN (Figure 5G).

### 2.7. The Regulatory Effect of CDN on EMT Markers and PI3K-Akt Pathway-Related Proteins

Based on the results of previous network pharmacology screening, machine learning validation, and molecular dynamics simulations, IGF1 has been identified as the core target of CDN in acting against IPF. The central role of the PI3K-AKT signaling pathway in the pathology of IPF is well-established, with IGF1 identified as a key upstream regulator. It is closely associated with the regulation of EMT and serves as an important molecular bridge linking epithelial injury with fibrosis progression. Therefore, subsequent experiments were conducted around the core hypothesis of “the CDN-IGF1/PI3K/AKT axis regulating EMT”, with a further exploration of the upstream roles of oxidative stress and inflammatory responses in this mechanism.

**Figure 6:**
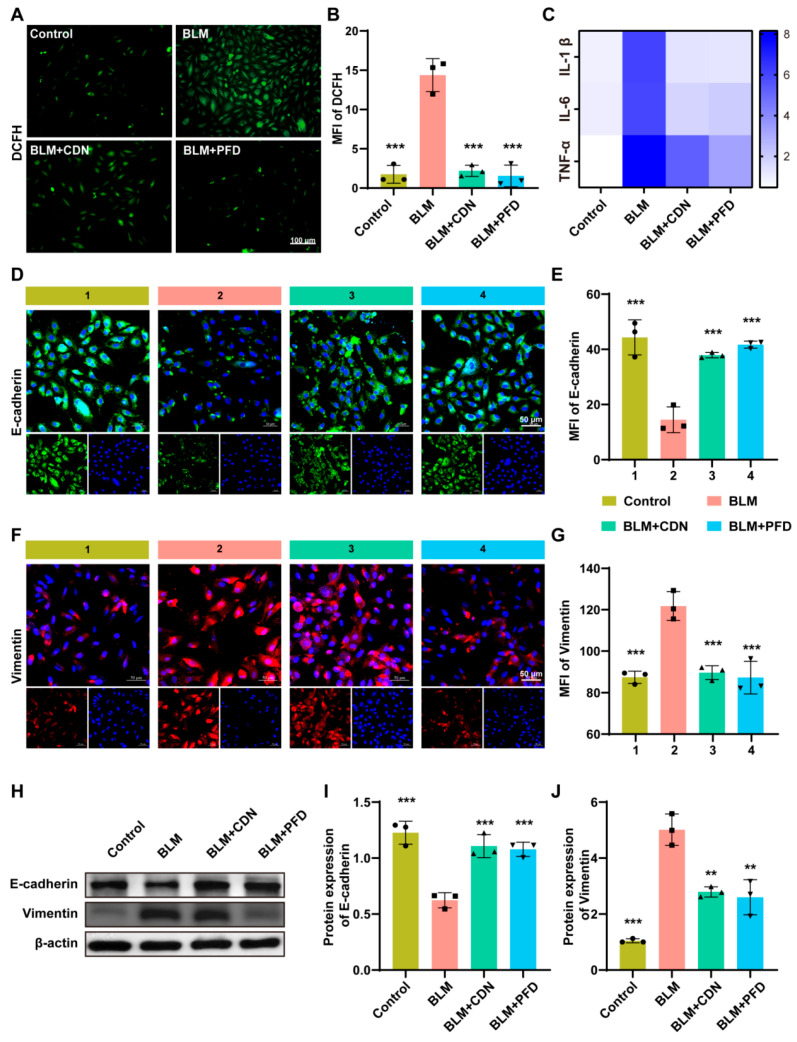
CND inhibits BLM-induced alveolar epithelial–mesenchymal transition in vitro. (**A**) Representative fluorescence images of DCFH-DA. Scale bar = 100 μm. (**B**) Quantification of the mean fluorescence intensity of DCFH. (**C**) Heatmap showing the mRNA expression levels of inflammatory cytokines IL-1β, IL-6, and TNF-α. The depth of color corresponds to the expression level. (**D**) Representative immunofluorescence staining results of E-cadherin. Scale bar = 50 μm. (**E**) Quantification of the mean fluorescence intensity of E-cadherin. (**F**) Representative immunofluorescence images results of vimentin. Scale bar = 50 μm. (**G**) Quantification of the mean fluorescence intensity of vimentin. The numbers 1, 2, 3, and 4 on the x-axis correspond to the Control, BLM only, BLM+CDN, and BLM+PFD groups, respectively. (**H**) Western blotting was used to detect E-cadherin, vimentin, and β-actin (internal reference control). (**I**,**J**) Standardized quantitative analysis of the protein expression levels of E-cadherin (**I**) and vimentin (**J**), with β-actin as the internal reference. ** *p* < 0.01, and *** *p* < 0.001.

The results showed that BLM treatment significantly increased the level of Reactive Oxygen Species (ROS) in A549 cells (as indicated by elevated DCFH-DA fluorescence intensity, Figure 6A,B) and elevated the transcript levels of the inflammatory cytokines IL-1β, IL-6, and TNF-α (Figure 6C). In contrast, CDN intervention significantly reversed these changes, and its effect was comparable to that of the positive control drug pirfenidone (PFD). This result is not an isolated antioxidant/anti-inflammatory effect—oxidative stress and chronic inflammation are known to be key upstream inducers of EMT: excessive ROS can trigger EMT by impairing epithelial cell barrier function, while inflammatory cytokines such as IL-6 and TNF-α can promote epithelial cell phenotypic transformation by activating downstream signaling pathways (e.g., the Signal Transducer and Activator of Transcription 3 (STAT3) pathway). The inhibitory effect of CDN on ROS and inflammatory cytokines essentially lays the foundation for the subsequent regulation of EMT by alleviating the “epithelial injury microenvironment”. After BLM induction, the cells demonstrated a significant downregulation of the epithelial marker E-cadherin (in both fluorescence and protein expression), in parallel with a notable upregulation of the mesenchymal marker vimentin (Figure 6D–J), indicating that the EMT process was activated. However, CDN treatment could dose-dependently reverse this trend, increasing the expression of E-cadherin and decreasing the expression of vimentin. This suggests that the inhibitory effect of CDN on EMT is the core phenotypic effect underlying its alleviation of BLM-induced pulmonary fibrosis, and the molecular mechanism of this effect requires further validation through the IGF1/PI3K-AKT pathway.

**Figure 7:**
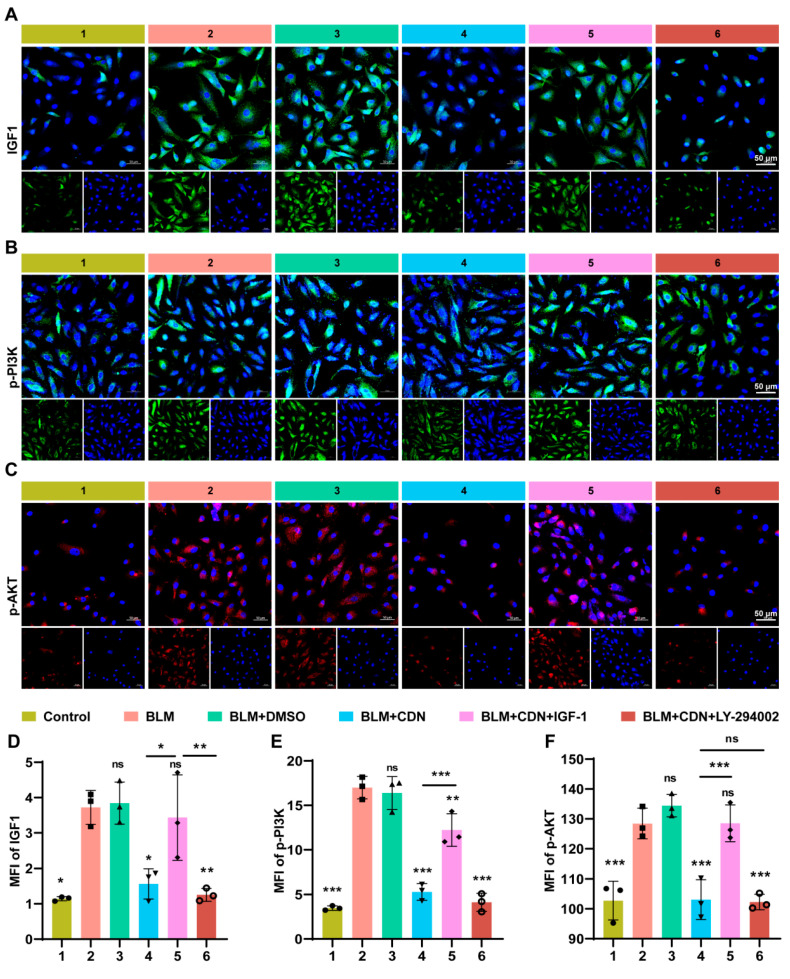
Validation of the IGF1/PI3K/AKT regulatory axis. (**A**–**C**) Representative immunofluorescence images of IGF1 (**A**), p-PI3K (**B**), and p-AKT (**C**). Treatment groups: 1, PBS (control); 2, BLM; 3, BLM + DMSO (vehicle control); 4, BLM + CDN; 5, BLM + CDN + IGF1; 6, BLM + CDN + LY294002 (a PI3K inhibitor). Scale bars: 50 μm. (**D**–**F**) Quantification of mean fluorescence intensity (MFI) for IGF1 (**D**), p-PI3K (**E**), and p-AKT (**F**) from immunofluorescence images. Data are presented as mean ± SD (*n* = 3 independent experiments); * *p* < 0.05, ** *p* < 0.01, *** *p* < 0.001; ns, not significant.

Verification experiments targeting the pathway showed that BLM treatment significantly upregulated the expression of IGF1, phosphorylated PI3K (p-PI3K), and phosphorylated AKT (p-AKT) (Figure 7A–F), confirming that the IGF1/PI3K-AKT pathway was abnormally activated in the IPF model. CDN intervention significantly inhibited the expression of these three molecules, directly targeting the activation link of this pathway. Further functional rescue experiments demonstrated that exogenous IGF1 supplementation could partially reverse the inhibitory effect of CDN on the pathway and its regulatory effect on EMT, while the PI3K-specific inhibitor LY294002 could enhance the aforementioned effects of CDN (Figure 7D–F). This series of results clarified the complete mechanism chain of “CDN inhibiting EMT by suppressing IGF1 activation, thereby downregulating the phosphorylation level of the PI3K-AKT pathway”. At the same time, it also explains why CDN can simultaneously regulate oxidative stress, inflammation, and EMT—the IGF1/PI3K-AKT pathway itself is a common downstream regulatory node for oxidative stress, inflammatory responses, and EMT. By targeting IGF1, the core upstream target of this pathway, CDN achieves multi-link intervention in the pathological process of IPF.

## 3. Discussion

This study systematically investigated the role and mechanism of CDN in IPF using an integrated approach encompassing network pharmacology, machine learning, molecular simulation, and in vitro experiments. Core findings demonstrated that CDN concentration-dependently restored viability in BLM-injured A549 cells and reduced the expression of fibrotic markers (α-SMA, fibronectin). Through multi-omics strategies, 6 core targets (including IGF1) were identified, with KEGG enrichment analysis highlighting the PI3K-Akt pathway as a key mediating pathway. Molecular docking confirmed stable binding between CDN and IGF1 (binding energy: −7.9 kcal/mol), and 100 ns molecular dynamics simulations further validated the stability of the CDN-IGF1 complex (mean binding free energy: −150.978 Kcal/mol). In vitro experiments further verified that CDN inhibits BLM-induced epithelial–mesenchymal transition (EMT) by suppressing the IGF1/PI3K/AKT axis—this inhibitory effect was reversed by exogenous IGF1 supplementation and enhanced by the PI3K inhibitor LY294002. These results align with CDN’s established anti-fibrotic roles in fibrosis [20,21], yet they reveal a distinct mechanism of action in pulmonary fibrosis: whereas CDN ameliorates liver and kidney fibrosis primarily via suppressing TGF-β or NF-κB cascades, its anti-fibrotic effect in BLM-induced alveolar epithelial injury appears to be mediated predominantly through the inhibition of IGF1/PI3K/AKT signaling [21]. While this study identifies the IGF1/PI3K/AKT axis as a critical target of CDN in BLM-induced alveolar epithelial injury, we acknowledge the central role of TGF-β signaling in IPF pathogenesis. Our computational screening did not prioritize TGF-β pathway components as top targets for CDN in this context, and the observed anti-fibrotic effects appear to be mediated primarily through IGF1 suppression. Whether CDN modulates TGF-β signaling in other IPF models or cell types remains an open question for future investigation. Additionally, CDN exhibits low cytotoxicity, with maximal anti-fibrotic efficacy at 20 µM and no toxicity observed up to 80 µM—consistent with its safety profile in hepatocytes [22,23]. While its anti-EMT effect (restoring E-cadherin expression and reducing Vimentin levels) mirrors that of other natural compounds such as berberine and resveratrol [24,25], CDN uniquely targets IGF1 as an upstream regulator of the PI3K/AKT axis, distinguishing its mechanism of action from other natural anti-fibrotic agents.

A critical advantage of CDN over conventional anti-IPF drugs lies in its food-derived origin. CDN is a major bioactive chalcone found in Zingiberaceae plants, including Alpinia katsumadai and Alpinia galanga, which are widely used as culinary spices in Southeast Asian and Indian diets [26,27]. This “food–medicine homology” property enables CDN to serve as a potential long-term nutritional intervention for IPF. The only FDA-approved IPF therapies, pirfenidone and nintedanib, merely slow disease progression without reversing fibrosis and are associated with significant adverse effects (pirfenidone causes photosensitivity and gastrointestinal distress, while nintedanib increases cardiovascular risk) [28,29]. Notably, CDN shares the “low toxicity + multi-target regulation” advantage of other food-derived anti-fibrotic compounds: Resveratrol (abundant in grapes and peanuts) mitigates BLM-induced pulmonary fibrosis in mice by regulating the SIRT1/AMPK metabolic axis [30], while curcumin (the active component of turmeric) alleviates IPF by modulating gut–lung axis metabolism [31]. Similarly, CDN’s inhibition of the IGF1/PI3K/AKT axis not only suppresses EMT but also potentially regulates cellular metabolism via mTOR (a downstream effector of PI3K/AKT that controls glucose uptake and protein synthesis [32].

Among the 6 identified core targets, IGF1 emerges as the primary mediator of CDN’s anti-IPF effects, supported by three lines of compelling evidence. First, from a functional perspective, the IGF1/PI3K/AKT axis is a well-recognized driver of EMT in IPF: binding of IGF1 to its receptor activates PI3K, leading to AKT phosphorylation and subsequent upregulation of EMT transcription factors, which directly downregulate the epithelial marker E-cadherin and upregulate the mesenchymal markers Vimentin and α-SMA [33,34]. In contrast, other algorithmically prioritized targets lack clear or direct links to alveolar epithelial-driven fibrosis and EMT, which was the core phenotypic focus of our investigation: CHRM3 primarily regulates airway smooth muscle contraction [35], TNNC1 is a cardiac troponin with no reported role in alveolar epithelial fibrosis [36], and MME/TEK are associated with angiogenesis [37]. While these targets may reflect broader systemic alterations in IPF, their mechanistic roles in the core EMT process remain less defined compared to the IGF1/PI3K/AKT axis. Second, from a molecular perspective, CDN exhibits the strongest binding affinity for IGF1 (−7.9 kcal/mol) compared to other core targets (e.g., CHRM3: −6.8 kcal/mol, TNNC1: −7.1 kcal/mol), and 100 ns molecular dynamics simulations confirmed the superior stability of the CDN-IGF1 complex (RMSD < 1 nm)—a critical prerequisite for effective target modulation [38]. Third, functional rescue experiments validated IGF1’s indispensability: exogenous IGF1 supplementation completely reversed CDN-induced suppression of PI3K/AKT phosphorylation and restoration of EMT markers. Collectively, these findings suggest that CDN may alleviate BLM-induced fibrosis in A549 cells by inhibiting the IGF1/PI3K/AKT axis. However, whether IGF1 is the exclusive or principal target of CDN requires further validation through genetic manipulation experiments.

This study also has several limitations that warrant consideration. First, all mechanistic conclusions are derived solely from the A549 human alveolar type II (ATII) epithelial cell line and have not yet been validated in primary alveolar type II cells or in vivo models. While A549 cells are widely used in IPF research due to their ability to recapitulate BLM-induced EMT and fibrotic responses [39,40,41], they are a malignant-transformed cell line with inherent differences from primary human ATII cells, such as robust surfactant synthesis [42,43]. To address these limitations and advance the translational potential of CDN as an IPF nutritional intervention, future studies will focus on three key directions: first, isolating primary human ATII cells from donor lungs (with ethical approval) to verify CDN’s effects on EMT, the IGF1/PI3K/AKT axis; second, conducting dietary intervention studies in BLM-induced IPF mice, with CDN supplemented in feed at doses of 100, 200, and 300 mg/kg to assess pulmonary fibrosis (via Ashcroft scoring of lung sections, hydroxyproline content, and fibrotic marker expression), and long-term safety (via body weight, food intake, and liver/kidney function markers [Alanine Aminotransferase (ALT), Aspartate Aminotransferase (AST), creatinine]); third, exploring pathway crosstalk by confirming direct binding between CDN and IGF1 via co-immunoprecipitation, and evaluating whether the anti-fibrotic effect of CDN depends on IGF1 signaling in conditional knockout mice or animal models treated with IGF1 receptor inhibitors.

## 4. Materials and Methods

### 4.1. Reagents

Key chemical reagents, including CDN (HY-N0279; 98.38%), BLM (HY-17565A; 99.77%), IGF-1 (HY-P7018), and LY-294002 (HY-10108; 99.86%), were all purchased from MedChemExpress (MCE, Monmouth Junction, NJ, USA). A549 cells were provided by Shanghai Zhongqiaoxinzhou Biotechnology Co., Ltd. (Shanghai, China). Penicillin-Streptomycin (100×) solution, DAPI and anti-fade mounting medium were sourced from Solarbio Science & Technology Co., Ltd. (Beijing, China). Dulbecco’s Modified Eagle Medium (DMEM) was supplied by Gibco (Grand Island, NY, USA) and the CCK-8 kit was provided by APExBIO (Houston, TX, USA). Other materials, namely a 4% paraformaldehyde solution and Phosphate Buffered Saline (PBS), were sourced from Shanghai Sunbiotech Co., Ltd. (Shanghai, China) and Sinozhongshuiqiao (Shanghai, China), respectively. Primary antibodies for immunofluorescence and Western blot analysis: Anti-α-SMA (ab5694, abcam, Cambridge, UK) for detection of α-smooth muscle actin (IF). Anti-fibronectin (sc-8422, Santa Cruz Biotechnology, Dallas, TX, USA) for detection of fibronectin (IF). Anti-E-cadherin (20874-1-AP, Proteintech, Rosemont, IL, USA) for detection of epithelial marker E-cadherin (IF, WB). Anti-Vimentin (10366-1-AP, Proteintech) for detection of mesenchymal marker vimentin (IF, WB). Anti-phospho-PI3K (AF3242, Affinity, Dallas, TX, USA) for detection of phosphorylated PI3K (IF). Anti-phospho-AKT (AP0274, ABclonal, Wuhan, China) for detection of phosphorylated AKT (IF). Anti-β-actin (#4970, Cell Signaling Technology, Danvers, Boston, MA, USA) was used as a loading control for Western blot analysis.

### 4.2. Cell Culture

A549 cells were cultured in Dulbecco’s Modified Eagle Medium (DMEM) supplemented with 10% fetal bovine serum (FBS) and 1% penicillin-streptomycin. Cells were maintained at 37 °C in a humidified atmosphere containing 5% CO_2_. As a widely used alveolar epithelial cell model, A549 cells are derived from human lung adenocarcinoma and retain alveolar type II-like properties, including surfactant production and responsiveness to fibrotic stimuli such as bleomycin (BLM), which supports their application in pulmonary fibrosis-related in vitro studies [39,42].

### 4.3. Cell Viability Analysis

The CCK-8 assay was used to evaluate the viability of A549 cells and determine the optimal CDN concentration for intervention. First, A549 cells were cultured in 96-well plates with a seeding number of 8 × 10^3^ cells per well (*n* = 6 per group) and cultured for 24 h to ensure attachment. Then, cells were treated with fresh medium supplemented with a gradient of CDN concentrations (0, 10, 20, 40, and 80 µM) or with BLM, and the incubation continued for another 24 h. Finally, following the preparation of a 1:9 mixture of CCK-8 reagent and DMEM, each well was supplemented with 100 μL of the solution. The plate was then placed in a 37 °C incubator for 2–4 h. Cell viability was assessed via determining the OD value at 490 nm with a microplate reader.

### 4.4. Immunofluorescence Analysis

After fixation with 4% paraformaldehyde, samples were incubated overnight with the primary antibody. Subsequently, samples were washed with PBS and then incubated with fluorescent secondary antibodies at 37 °C for 2 h, followed by DAPI staining to label cell nuclei. Fluorescent images were captured using a confocal laser scanning microscope (LSM800, Zeiss, Oberkochen, Germany).

### 4.5. Target Screening of CDN

We initiated the screening by obtaining the chemical structure and SMILES of CDN from PubChem (https://pubchem.ncbi.nlm.nih.gov/) (accessed on 6 March 2025). We then employed four databases—SwissTargetPrediction (http://www.swisstargetprediction.ch) (accessed on 6 March 2025), ETCM (http://www.tcmip.cn/ETCM/) (accessed on 6 March 2025), PharmMapper (http://www.lilab-ecust.cn/pharmmapper/) (accessed on 7 March 2025), and STITCH (https://stitch.embl.de/) (accessed on 7 March 2025)—to predict its direct targets. The species filter was set as Homo sapiens exclusively. The UniProt database (https://www.uniprot.org/) (accessed on 7 March 2025) was utilized to normalize the collected drug target names into official gene symbols, ensuring the relevance and accuracy of subsequent analyses. This data processing step involved consolidating all predictions, converting target identifiers to standardized gene names, and systematically applying filters to exclude non-human proteins, invalid entries, and duplicates, thereby generating a final refined and non-redundant list of human gene targets.

### 4.6. Target Screening for Idiopathic Pulmonary Fibrosis

For the retrieval of targets associated with IPF, we obtained information from five public databases as follows: First, we searched the GeneCards database using the keyword “idiopathic pulmonary fibrosis” and set a relevance score threshold of ≥7. Subsequently, we queried the Online Mendelian Inheritance in Man (OMIM) database with the same keyword, retaining only entries with a phenotypic relationship grade of 3 (indicating a direct phenotypic association) in the Pheno-Map table. Additionally, we consulted the MalaCards database (Entry ID: INT457; OMIM ID: 178500) and selected genes with a relevance score of ≥7 from it. Meanwhile, we accessed the Comparative Toxicogenomics Database (CTD), restricting the evidence type to “direct evidence” and the association type to only “marker/mechanism” or “therapeutic”. This screening process resulted in 22 high-confidence genes. At the same time, the Genome-Wide Association Study (GWAS) Catalog was interrogated for IPF-associated genetic loci. The applied filtering criteria required genome-wide significance (*p*-value ≤ 5 × 10^−8^) and gene annotation for all retained single nucleotide polymorphisms (SNPs). Additionally, we used “Idiopathic Pulmonary Fibrosis” and “Homo sapiens” as keywords to extract data related to IPF from four datasets—GSE24206, GSE150910, GSE213001, and GSE124685—housed in the GEO database (https://www.ncbi.nlm.nih.gov/geo/) (accessed on 12 April 2025). First, we applied the ComBat method to the merged datasets for batch correction, aiming to eliminate technical biases among different datasets. Subsequently, we performed inter-group differential gene expression analysis using the Limma package, with the screening criteria set as |logFC| > 1 and a *p*-value < 0.05 after false discovery rate (FDR) correction. By integrating the analysis results of all the aforementioned datasets, we finally identified key genes closely associated with idiopathic pulmonary fibrosis.

### 4.7. Construction of Protein–Protein Interaction (PPI) Network and Functional Enrichment of Target Genes

The Venn diagram of CDN targets and genes related to IPF was created using an online tool (http://bioinformatics.psb.ugent.be/webtools/Venn/) (accessed on 22 April 2025) for visualization. One hundred and ninety-three overlapping genes were uploaded to the STRING database (version 11.5, https://cn.string-db.org/) (accessed on 22 April 2025) with the species set to “Homo sapiens” to generate a PPI network. The network was visualized using Cytoscape 3.10.2, and the topological parameters (Degree) of these targets were obtained using the NetworkAnalyst tool in Cytoscape 3.10.2. The core target-related information was entered into the DAVID 6.8 database (available at https://david.ncifcrf.gov/) (accessed on 24 April 2025), where the identifier was configured as “OFFICIAL GENE SYMBOL” and “Homo sapiens” was selected as the corresponding species for performing GO and KEGG pathway analyses. The GO analysis annotated gene functions through three levels: cellular components (CC), biological processes (BP), and molecular functions (MF), with *p* < 0.05 as the threshold for enrichment analysis results visualization.

### 4.8. Machine Learning

A multi-model computational framework was implemented to identify core genes associated with IPF. The analysis integrated five supervised machine learning algorithms—k-Nearest Neighbors (KNN), LASSO regression, artificial neural network (ANN), random forest (RF), and support vector machine (SVM). All modeling was performed in R (v4.3.1) utilizing the randomForest, caret, glmnet, nnet, e1071, pROC, and tidyverse packages [44]. The dataset was imported in CSV format with sample IDs set as row names and gene symbols retained using the check.names = FALSE parameter to avoid syntax alterations, then split into features (all columns except the last, representing gene expression profiles) and labels (last column, indicating IPF status); labels were converted to factors and standardized via make.names(labels) to resolve syntax conflicts, followed by a stratified 80:20 training–test split using createDataPartition(y, *p* = 0.8, list = FALSE, seed = 123) to preserve class distribution, resulting in a training set (96 samples: 64 IPF cases, 32 controls) and a test set (24 samples: 16 IPF cases, 8 controls). All models were trained under 5-fold cross-validation (via trainControl(method = “cv”, number = 5, classProbs = TRUE)), with feature scaling (centering and standardization) applied to KNN, ANN, and SVM models using preProcess = c(“center”, “scale”), and hyperparameters optimized to maximize classification performance: KNN via tuneLength = 10 (grid search) to select optimal k (number of neighbors), LASSO with alpha = 1 and lambda ranging from 0.001 to 0.1 (20 equidistant values), ANN with hidden layer size of 5/10/15 and weight decay of 0.001/0.01/0.1 (via tuneGrid) and trace = FALSE, RF with ntree = 500 and mtry = sqrt(ncol(X_train)), and SVM using radial basis function (RBF) kernel (svmRadial) with hyperparameters (gamma, cost) optimized via tuneLength = 10. Variable importance was extracted for core gene selection: RF via mean decrease in Gini impurity, LASSO via absolute regression coefficients, and KNN/SVM/ANN via permutation importance (decrease in AUC-ROC after random shuffling). Scores were standardized to 0–1 for cross-model comparison, a mean importance score was calculated for each gene, and core targets were defined as the top 6 genes with the highest mean importance (stable across ≥4 models). Model performance was assessed on the test set using AUC-ROC, accuracy, and Kappa coefficients, with ROC curves generated via the pROC package and metrics visualized via dotplot()/bwplot() from caret—all models achieved AUC > 0.95, confirming strong discriminative ability for IPF-related gene signatures.

### 4.9. Molecular Docking Validation

The mol2 format file of CDN was downloaded from the TCMSP database and converted to a PDB format file via OpenBabel 2.4.1 software. From the UniProt database, the PDB IDs corresponding to the 6 core targets with the lowest resolution values were screened. Subsequently, the PDB files of the corresponding protein structures were downloaded from the PDB database, and molecular docking was performed via AutoDockTools 1.5.7 software. Finally, the molecular docking results were visualized via PyMOL 2.0 software.

### 4.10. Molecular Dynamics Simulations

All MD simulations were carried out in GROningen MAchine for Chemical Simulations (GROMACS) 2022. The CHARMM36 force field was applied to the receptor protein, while ligand parameters were derived from the GAFF2 force field via the Automated Force Field Topology Builder (AutoFF) website, with RESP-derived atomic charges assigned using Antechamber. The system was solvated in a TIP3P water model within a 1 nm cubic box and neutralized with 0.15 M NaCl under physiological ion concentration. A Particle Mesh Ewald (PME) cutoff of 1.0 nm was applied to handle long-range electrostatic interactions, and bonds were constrained with the LINCS algorithm. Energy minimization was performed using a combination of steepest descent (3000 steps) and conjugate gradient (2000 steps) methods, incorporating sequential constraint releases: initially on solute, then on counterions, and finally with all atoms unconstrained. A 100 ns production MD simulation was conducted under Isothermal–Isobaric (NPT) conditions using a 2 fs integration time step. The temperature was maintained at 310 K with the Nosé–Hoover thermostat, and the pressure was held at 1 bar using the Parrinello–Rahman barostat. Trajectories were analyzed for RMSD, RMSF, hydrogen bonds, Rg, and SASA using built-in GROMACS tools. A suite of analyses, including RMSD, RMSF, hydrogen bonding, Rg, and SASA, was performed on the trajectories using GROMACS utilities. Binding free energy calculations were performed utilizing the MM-PBSA approach with the g_mmpbsa package.

### 4.11. RNA Extraction and Real-Time Quantitative PCR

Total RNA was extracted from samples using TRIzol reagent (Invitrogen, Carlsbad, CA, USA) and quantified using a NanoDrop spectrophotometer (Thermo Fisher Scientific, Waltham, MA, USA). cDNA was synthesized from 2 µg of RNA using the PrimeScript RT reagent kit (Takara Bio, Kyoto, Japan) according to the manufacturer’s instructions. Real-time quantitative PCR was performed using SYBR Green Premix (Takara Bio) on a QuantStudio 5 system (Applied Biosystems, Foster City, CA, USA). The PCR protocol was as follows: initial denaturation at 95 °C for 3 min; followed by 40 cycles of denaturation at 95 °C for 15 s, annealing at 60 °C for 30 s, and extension at 72 °C for 30 s. GAPDH was used as the internal reference gene, and the relative expression levels of target genes were calculated using the 2^−ΔΔCt^ method.

### 4.12. Western Blot Analysis

Total proteins were extracted from cultured cells by lysing the samples in a protein extraction buffer containing protease inhibitors, phosphatase inhibitors, and phenylmethylsulfonyl fluoride (PMSF) (kit sourced from KeyGEN, Nanjing, China). Proteins were first separated using SDS-PAGE. Following electrophoresis, the resolved proteins from the gel were blotted onto polyvinylidene fluoride (PVDF) membranes. The PVDF membranes were blocked with 5% skimmed milk (Cat. No. 232100, obtained from BD, Franklin Lakes, NJ, USA) at room temperature for 2 h. Following this, membranes were probed with primary antibodies in an overnight incubation at 4 °C. Subsequently, the membranes were washed with PBST buffer, after which goat anti-mouse secondary antibody (sourced from ZSGBBIO, Beijing, China) was added, followed by incubation at room temperature for 2 h. Finally, protein bands were developed using an enhanced chemiluminescent solution (obtained from Bio-Rad Laboratories, Hercules, CA, USA), and quantitative analysis was conducted using Image Lab 3.0. The results were ultimately normalized using β-actin as the internal reference.

### 4.13. Quantitative and Statistical Analysis

All experiments were independently repeated at least 3 times to ensure reproducibility. Data are expressed as mean ± standard deviation (SD). Statistical differences between two groups were analyzed using the unpaired Student’s *t*-test with a two-tailed distribution. Differences between data from more than two groups were analyzed by one-way analysis of variance (ANOVA) followed by Bonferroni’s post hoc test (GraphPad Prism 8.0, San Diego, CA, USA). ** Statistically significant differences were defined as a *p*-value < 0.05. Significant differences in all figures are denoted as follows: * *p* < 0.05, ** *p* < 0.01, *** *p* < 0.001.

## 5. Conclusions

This study systematically investigated the role and mechanism of CDN in IPF via an integrated approach of network pharmacology, machine learning, molecular simulation, and in vitro experiments. Key findings showed that CDN’s core IPF-related target (e.g., IGF1) was identified; molecular docking and dynamics confirmed stable CDN-IGF1 binding; and in vitro experiments verified CDN alleviated BLM-induced A549 cell fibrosis and EMT by inhibiting the IGF1/PI3K/AKT axis. These results suggest that CDN may alleviate BLM-induced fibrosis in A549 cells via the IGF1/PI3K/AKT axis. While these findings provide a preliminary mechanistic insight and a scientific basis for CDN as a potential therapeutic candidate, further validation in primary cells and in vivo models is necessary to assess its translational potential. Future studies will further confirm its translational value, supporting the development of natural product-based IPF treatments.

## Data Availability

The original contributions presented in this study are included in the article/Supplementary Materials. Further inquiries can be directed to the corresponding authors.

## Supplementary Materials

The following supporting information can be downloaded at: https://www.mdpi.com/article/10.3390/ijms27010249/s1.

## References

[1] Lederer D.J., Martinez F.J. (2018). Idiopathic Pulmonary Fibrosis. N. Engl. J. Med..

[2] Maher T.M., Bendstrup E., Dron L., Langley J., Smith G., Khalid J.M., Patel H., Kreuter M. (2021). Global Incidence and Prevalence of Idiopathic Pulmonary Fibrosis. Respir. Res..

[3] Lancaster L.H., de Andrade J.A., Zibrak J.D., Padilla M.L., Albera C., Nathan S.D., Wijsenbeek M.S., Stauffer J.L., Kirchgaessler K.U., Costabel U. (2017). Pirfenidone Safety and Adverse Event Management in Idiopathic Pulmonary Fibrosis. Eur. Respir. Rev..

[4] Ahangari F., Becker C., Foster D.G., Chioccioli M., Nelson M., Beke K., Wang X., Justet A., Adams T., Readhead B. (2022). Saracatinib, a Selective Src Kinase Inhibitor, Blocks Fibrotic Responses in Preclinical Models of Pulmonary Fibrosis. Am. J. Respir. Crit. Care Med..

[5] Wang Z., Xu G., Gao Y., Zhan X., Qin N., Fu S., Li R., Niu M., Wang J., Liu Y. (2019). Cardamonin from a Medicinal Herb Protects against Lps-Induced Septic Shock by Suppressing Nlrp3 Inflammasome. Acta Pharm. Sin. B.

[6] Heydarian A., Tahvilian N., Shahinfar H., Abbas-Hashemi S.A., Daryabeygi-Khotbehsara R., Aryaeian N. (2024). Effect of Cardamom Consumption on Inflammation and Blood Pressure in Adults: A Systematic Review and Meta-Analysis of Randomized Clinical Trials. Food Sci. Nutr..

[7] Delgadillo-Puga C., Torre-Villalvazo I., Cariño-Cervantes Y.Y., García-Luna C., Soberanes-Chávez P., de Gortari P., Noriega L.G., Bautista C.J., Cisneros-Zevallos L. (2023). Cardamom (*Elettaria cardamomum* (L.) Maton) Seeds Intake Increases Energy Expenditure and Reduces Fat Mass in Mice by Modulating Neural Circuits That Regulate Adipose Tissue Lipolysis and Mitochondrial Oxidative Metabolism in Liver and Skeletal Muscle. Int. J. Mol. Sci..

[8] Jin J., Qiu S., Wang P., Liang X., Huang F., Wu H., Zhang B., Zhang W., Tian X., Xu R. (2019). Cardamonin Inhibits Breast Cancer Growth by Repressing HIF-1α-Dependent Metabolic Reprogramming. J. Exp. Clin. Cancer Res..

[9] Liao Q., Shi D.H., Zheng W., Xu X.J., Yu Y.H. (2010). Antiproliferation of Cardamonin Is Involved in Mtor on Aortic Smooth Muscle Cells in High Fructose-Induced Insulin Resistance Rats. Eur. J. Pharmacol..

[10] Chen H., Shi D., Niu P., Zhu Y., Zhou J. (2018). Anti-Inflammatory Effects of Cardamonin in Ovarian Cancer Cells Are Mediated Via Mtor Suppression. Planta Medica.

[11] Lu S., Lin C., Cheng X., Hua H., Xiang T., Huang Y., Huang X. (2018). Cardamonin Reduces Chemotherapy Resistance of Colon Cancer Cells Via the Tsp50/Nf-Κb Pathway In Vitro. Oncol. Lett..

[12] Tie D., He M., Li W., Xiang Z. (2025). Advances in the Application of Network Analysis Methods in Traditional Chinese Medicine Research. Phytomedicine.

[13] Greener J.G., Kandathil S.M., Moffat L., Jones D.T. (2022). A Guide to Machine Learning for Biologists. Nat. Rev. Mol. Cell Biol..

[14] Tan L.H., Kwoh C.K., Mu Y. (2024). Rmsdxna: Rmsd Prediction of Nucleic Acid-Ligand Docking Poses Using Machine-Learning Method. Brief. Bioinform..

[15] Kihn K.C., Purdy O., Lowe V., Slachtova L., Smith A.K., Shapiro P., Deredge D.J. (2024). Integration of Hydrogen-Deuterium Exchange Mass Spectrometry with Molecular Dynamics Simulations and Ensemble Reweighting Enables High Resolution Protein-Ligand Modeling. J. Am. Soc. Mass Spectrom..

[16] Kuzmanic A., Zagrovic B. (2010). Determination of Ensemble-Average Pairwise Root Mean-Square Deviation from Experimental B-Factors. Biophys. J..

[17] Bezerra I.C., de Oliveira Viana J., Weber K.C., Gubert P. (2025). Dynamispectra: A Python Software Package and Web Platform for Molecular Dynamics Data Analysis in Computational Biology. J. Chem. Inf. Model..

[18] Yu C., Ma L., Li K., Li S., Liu Y., Zhou Y., Yan D. (2016). Molecular Dynamics Simulation Studies of Hyperbranched Polyglycerols and Their Encapsulation Behaviors of Small Drug Molecules. Phys. Chem. Chem. Phys..

[19] Venkateswarlu D. (2014). Structural Insights into the Interaction of Blood Coagulation Co-Factor Viiia with Factor Ixa: A Computational Protein-Protein Docking and Molecular Dynamics Refinement Study. Biochem. Biophys. Res. Commun..

[20] Zhang B., Chen Z.Y., Jiang Z., Huang S., Liu X.H., Wang L. (2023). Nephroprotective Effects of Cardamonin on Renal Ischemia Reperfusion Injury/Uuo-Induced Renal Fibrosis. J. Agric. Food Chem..

[21] Ye Z., Niu Z., Li J., Li Z., Hu Y. (2024). Cardamonin Inhibits Silicosis Development through the Pi3k-Akt Signaling Pathway. Ecotoxicol. Environ. Saf..

[22] Alkhalifah E.A.R., Alobaid A.A., Almajed M.A., Alomair M.K., Alabduladheem L.S., Al-Subaie S.F., Akbar A., Attimarad M.V., Younis N.S., Mohamed M.E. (2022). Cardamom Extract Alleviates the Oxidative Stress, Inflammation and Apoptosis Induced during Acetaminophen-Induced Hepatic Toxicity via Modulating Nrf2/HO-1/NQO-1 Pathway. Curr. Issues Mol. Biol..

[23] Atef Y., El-Fayoumi H.M., Abdel-Mottaleb Y., Mahmoud M.F. (2017). Effect of Cardamonin on Hepatic Ischemia Reperfusion Induced in Rats: Role of Nitric Oxide. Eur. J. Pharmacol..

[24] Ma Z., Zhu L., Wang S., Guo X., Sun B., Wang Q., Chen L. (2022). Berberine Protects Diabetic Nephropathy by Suppressing Epithelial-to-Mesenchymal Transition Involving the Inactivation of the Nlrp3 Inflammasome. Ren. Fail..

[25] Qian Y., Wang R., Wei W., Wang M., Wang S. (2021). Resveratrol Reverses the Cadmium-Promoted Migration, Invasion, and Epithelial-Mesenchymal Transition Procession by Regulating the Expression of Zeb1. Hum. Exp. Toxicol..

[26] Yamamoto N., Kawabata K., Sawada K., Ueda M., Fukuda I., Kawasaki K., Murakami A., Ashida H. (2011). Cardamonin Stimulates Glucose Uptake through Translocation of Glucose Transporter-4 in L6 Myotubes. Phytother. Res..

[27] Ashokkumar K., Murugan M., Dhanya M.K., Warkentin T.D. (2020). Botany, Traditional Uses, Phytochemistry and Biological Activities of Cardamom [*Elettaria cardamomum* (L.) Maton]—A Critical Review. J. Ethnopharmacol..

[28] Lancaster L., Morrison L., Auais A., Ding B., Iqbal A., Polman B., Flaherty K.R. (2017). Safety of Pirfenidone in Patients with Idiopathic Pulmonary Fibrosis: Experience from 92 Sites in an Open-Label Us Expanded Access Program. Pulm. Ther..

[29] Noth I., Wijsenbeek M., Kolb M., Bonella F., Moros L., Wachtlin D., Corte T.J. (2019). Cardiovascular Safety of Nintedanib in Subgroups by Cardiovascular Risk at Baseline in the Tomorrow and Inpulsistrials. Eur. Respir. J..

[30] Li H., Wang X., Deng Y., Liu M., Li W., Wang J., Zeng C., Dai H. (2025). Resveratrol Alleviates Lipopolysaccharide-Induced Acute Lung Injury through Blocking the Excessive Autophagy/Mitophagy Via Sirt1/Pgc-1α and Tnf/Nf-Κb/Jnk Pathways. Int. J. Biol. Macromol..

[31] Miao Y.M., Zhang Y.J., Qiao S.M., Xia Y.F., Wei Z.F., Dai Y. (2021). Oral Administration of Curcumin Ameliorates Pulmonary Fibrosis in Mice through 15d-Pgj2-Mediated Induction of Hepatocyte Growth Factor in the Colon. Acta Pharmacol. Sin..

[32] Zhou X., Zhou R., Li Q., Jie X., Hong J., Zong Y., Dong X., Zhang S., Li Z., Wu G. (2019). Cardamonin Inhibits the Proliferation and Metastasis of Non-Small-Cell Lung Cancer Cells by Suppressing the Pi3k/Akt/Mtor Pathway. Anti-Cancer Drugs.

[33] Kheirollahi V., Khadim A., Kiliaris G., Korfei M., Barroso M.M., Alexopoulos I., Vazquez-Armendariz A.I., Wygrecka M., Ruppert C., Guenther A. (2022). Transcriptional Profiling of Insulin-Like Growth Factor Signaling Components in Embryonic Lung Development and Idiopathic Pulmonary Fibrosis. Cells.

[34] Mei Q., Liu Z., Zuo H., Yang Z., Qu J. (2021). Idiopathic Pulmonary Fibrosis: An Update on Pathogenesis. Front. Pharmacol..

[35] Wain L.V., Shrine N., Artigas M.S., Erzurumluoglu A.M., Noyvert B., Bossini-Castillo L., Obeidat M., Henry A.P., Portelli M.A., Hall R.J. (2017). Genome-Wide Association Analyses for Lung Function and Chronic Obstructive Pulmonary Disease Identify New Loci and Potential Druggable Targets. Nat. Genet..

[36] Jordan E., Peterson L., Ai T., Asatryan B., Bronicki L., Brown E., Celeghin R., Edwards M., Fan J., Ingles J. (2021). Evidence-Based Assessment of Genes in Dilated Cardiomyopathy. Circulation.

[37] Mhatre I., Abdelhalim H., Degroat W., Ashok S., Liang B.T., Ahmed Z. (2023). Functional Mutation, Splice, Distribution, and Divergence Analysis of Impactful Genes Associated with Heart Failure and Other Cardiovascular Diseases. Sci. Rep..

[38] Verma J., Vashisth H. (2024). Structural Models for a Series of Allosteric Inhibitors of Igf1r Kinase. Int. J. Mol. Sci..

[39] Chen K.J., Li Q., Wen C.M., Duan Z.X., Zhang J.Y., Xu C., Wang J.M. (2016). Bleomycin (Blm) Induces Epithelial-to-Mesenchymal Transition in Cultured A549 Cells Via the Tgf-Β/Smad Signaling Pathway. J. Cancer.

[40] Ma Z., Ma C., Zhang Q., Bai Y., Mu K., Liu X., Yang Q. (2021). Role of Cxcl16 in Blm-Induced Epithelial-Mesenchymal Transition in Human A549 Cells. Respir. Res..

[41] Muthuramalingam K., Cho M., Kim Y. (2020). Cellular Senescence and Emt Crosstalk in Bleomycin-Induced Pathogenesis of Pulmonary Fibrosis-an in Vitro Analysis. Cell Biol. Int..

[42] Smith B.T. (1977). Cell Line A549: A Model System for the Study of Alveolar Type Ii Cell Function. Am. Rev. Respir. Dis..

[43] Öğünç Keçeci Y., İncesu Z. (2022). Mitochondrial Oxidative Phosphorylation Became Functional under Aglycemic Hypoxia Conditions in A549 Cells. Mol. Biol. Rep..

[44] Stretch C., Khan S., Asgarian N., Eisner R., Vaisipour S., Damaraju S., Graham K., Bathe O.F., Steed H., Greiner R. (2013). Effects of Sample Size on Differential Gene Expression, Rank Order and Prediction Accuracy of a Gene Signature. PLoS ONE.

