# Supplementary material for: Integrating Network Pharmacology, Machine Learning, and Experimental Validation to Elucidate the Mechanism of Cardamonin in Treating Idiopathic Pulmonary Fibrosis
Source: Int J Mol Sci. 2025 Dec 25;27(1):249. doi: 10.3390/ijms27010249 (PMC12786256; doi:10.3390/ijms27010249)
Supplement: Supplementary file 1 [file ijms-27-00249-s001.zip › ijms-3980623-supplementary/Figure S1.pdf]

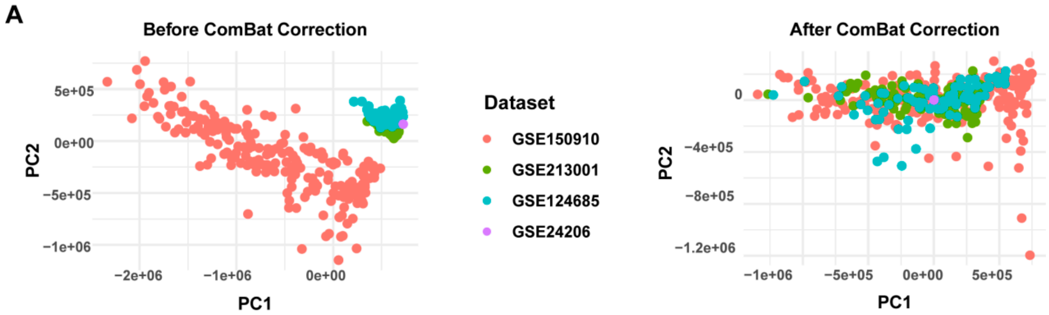

Figure **S1.** Principal component analysis (PCA) plots of gene expression data from four independent datasets (GSE150910, GSE213001, GSE124685, GSE24206). (**A**) Before ComBat Correction. (**B**) After ComBat Correction.
