# Supplementary material for: Integrating Network Pharmacology, Machine Learning, and Experimental Validation to Elucidate the Mechanism of Cardamonin in Treating Idiopathic Pulmonary Fibrosis
Source: Int J Mol Sci. 2025 Dec 25;27(1):249. doi: 10.3390/ijms27010249 (PMC12786256; doi:10.3390/ijms27010249)
Supplement: Supplementary file 1 [file ijms-27-00249-s001.zip › ijms-3980623-supplementary/Table S1.pdf]

Table S1 Potential Targets of CDN

Database	Jene symble
**ETCM**	KCNA3
	TYR
	TUBB1
	TNFRSF1A
	SENP6
	PTPN1
	PSMB5
	PPARG
	PDPK1
	P4HB
	NR1H4
	NLRP3
	HSD17B2
	HSD17B1
	F3
	CYP19A1
	CHRNA7
	BACE1
	AR
	APP
	ALOX5
	ABCG2
**pharmmapper**	CA2
	CES1
	MAPK14
	ESR1

	BCHE
	GSTP1
	CDK5R1
	PDE5A
	AKR1B1
	CDK2
	CHEK1
	PIM1
	LTA4H
	IMPA1
	HSPA8
	HCK
	CDK6
	HSP90AA1
	EGFR
	NQO1
	ANG
	FGFR1
	CFB
	BCAT2
	MAPK10
	MAOB
	MAPK8
	AMY1A
	AMY1B
	AMY1C
	Esr2
	MTHFD1
	F2

	DHFR
	CRAT
	CFD
	PNP
	MIF
	PLAU
	GSK3B
	CCNA2
	PYGL
	PGR
	PCK1
	DAPK1
	MMP8
	ADAM17
	ABO
	LGALS7
	LGALS7B
	KDR
	UCK2
	NOS3
	DCK
	AURKA
	ISG20
	CBR1
	KIF11
	AHCY
	CDA
	AKR1C1
	TTR

	CCNT1
	HK1
	SHBG
	HSD11B1
	ALB
	CYP2C9
	AMD1
	REG1A
	F10
	PAH
	PDE4D
	OTC
	EPHB4
	IMPDH2
	EPHX2
	MMP3
	SULT2A1
	ALDH2
	TGM2
	TNK2
	RHEB
	CASP3
	PARP1
	AKR1C3
	CTNNA1
	PDE4B
	HMGCR
	DHODH
	SRC

	ADH5
	PLA2G10
	FGFR2
	MMP7
	SOD2
	REN
	TGFBR1
	PNMT
	PRKACA
	LCK
	MMP13
	SYK
	MTAP
	ACP3
	NOS2
	RNASE4
	IGF1R
	THRB
	HDAC8
	ITK
	IL2
	TPI1
	SSE1
	RNASE3
	FABP4
	DPP4
	HEXB
	FHIT
	BIRC7

	B3GAT1
	NR1H3
	UMPS
	ESRRG
	NR1H2
	BST1
	PADI4
	ALDOA
	FKBP1A
	MET
	PLAT
	CCL5
	TGM3
	ELANE
	TYMS
	ERBB4
	NR3C2
	RXRA
	PDK2
	GSR
	RAB11A
	PPARA
	FECH
	NR1I2
	PPCDC
	JAK2
	CTSS
	LGALS2
	S100A9

	MME
	FABP6
	CBS
	SHMT1
	PAK6
	GSTT2B
	MAPKAPK2
	CLK1
	FNTA
	SULT2B1
	MAN1B1
	CTSF
	IGF1
	GPI
	SERPINA1
	CTSK
	TPH1
	MMP12
	XIAP
	LGALS3
	PAPSS1
	BLVRB
	CTSG
	ABL1
	HAGH
	SDS
	MMP9
	SULT1E1
	RAB5A

	F11
	ADK
	ACADM
	THRA
	PITPNA
	LCN2
	SRM
	PLA2G2A
	KIT
	GSTA1
	VDR
	LYZ
	F7
	EIF4E
	OAT
	GSTM1
	DPEP1
	BHMT
	APRT
	HRAS
	ARG1
	FDPS
	NR3C1
	TPSB2
	CHIT1
	GART
	HINT1
	RARA
	KAT2B

	GSTM2
	DTYMK
	FOLH1
	MAPK1
	STAT1
	PIK3R1
	CMA1
	GNPDA1
	HMOX1
	GSTA3
	PPP1CC
	TGFB2
	GALE
	ARL5A
	GP1BA
	MMP16
	AMY2A
	HPRT1
	IMPDH1
	MAP2K1
	NT5M
	ATIC
	RAB9A
	ADAM33
	TEK
	FKBP1B
	NMNAT3
	CD209
	CTSB

	RNASE2
	CDC42
	SELE
	FGG
	PKLR
	GRB2
	RAF1
	CASP1
	GMPR
	SEC14L2
	RARG
	ERI1
	HNMT
	DUT
	EEA1
	TRDMT1
	FABP3
	GMPR2
	ITPKA
	NME2
	HADH
	FKBP3
	BCL2L1
	RND3
	UAP1
	DOT1L
	RAP2A
	RXRB
	WARS1

	GSTZ1
	Pfkfb1
	APAF1
	DCXR
	INSR
	AK1
	TAP1
	RAN
	DCPS
	RAC1
	Arl5b
	RFK
	BTK
	IVD
	ME2
	AKT1
	SULT1A1
	TTPA
	CRYZ
	NDST1
	SPR
**STITCH**	cardamonin
	SNAI1
	VEGFA
	RPS6KB1
**superpred**	APEX1
	KLF5
	TRIM24
	TDP1

	NFKB1
	ALOX12
	NFE2L2
	RORB
	SLC6A5
	PSMB1
	CTSD
	SLC2A1
	CHRM4
	HSD17B10
	TOP2A
	MDM4
	CHRM5
	HTR2C
	PIN1
	ADAM10
	PDGFRA
	CSNK2B
	GLS
	DPP9
	ITGB1
	CHRNB4
	GBA2
	DRD1
	PSMB2
	CYP3A4
	CACNA1B
	ERAP1
	HDAC7

	GUSB
	GRK5
	GRIN1
	XDH
	HDAC2
	HDAC5
	C5AR1
	FGR
	CHRM3
	GLRA1
	FPR2
	CCR1
	GABRA1
	P2RY6
	CDK1
	SAE1
	CHRNA1
	CDK5
	AVPR1B
	PRCP
	PTPN11
	DPP8
	HDAC9
	BRDT
	CLK4
	FPR1
	GPBAR1
	KEAP1
	NR4A1

	SERPINE1
	PDGFRB
	ACACB
**Swiss TargetPrediction**	PTGS2
	ABCB1
	SLC22A12
	TLR9
	MAOA
	TERT
	PTGS1
	MCL1
	ODC1
	FLT3
	HSP90B1
	HSPA1A
	CXCR4
	GCGR
	GPR84
	ALPL
	MMP1
	CCR4
	CXCR2
	PDE10A
	MAPKAPK5
	ALK
	PRKAA1
	CTSL
	NOX4
	HDAC1

	GPR35
	CXCR1
	EGLN1
	ALOX15
	HSP90AB1
	KCNMA1
	STS
	TNNC1
	TNNT2
	TNNI3
	SNCA
	PRKCA
	PRKCB
	PRKCE
	HRH3
	GRK6
	IGFBP3
	PTGER4
	PTGER2
	PTGER3
	ACHE
	KDM4E
	NTRK2
	CCNB3
	CCNB1
	CCNB2
	FLT1
	FLT4
	AURKC

	FEN1
	EDNRA
	AVPR2
	IDH1
	MAPT
	HDAC3
	HDAC6
	PTK2
	PLK1
	PTGS2
	ABCB1
	SLC22A12
	TLR9
	MAOA
	TERT
	PTGS1
	MCL1
	ODC1
	FLT3
	HSP90B1
	HSPA1A
	CXCR4
	GCGR
	GPR84
	ALPL
	MMP1
	CCR4
	CXCR2
	PDE10A

	MAPKAPK5
	ALK
	PRKAA1
	CTSL
	NOX4
	HDAC1
	GPR35
	CXCR1
	EGLN1
	ALOX15
	HSP90AB1
	KCNMA1
	STS
	TNNC1
	TNNT2
	TNNI3
	SNCA
	PRKCA
	PRKCB
	PRKCE
	HRH3
	GRK6
	IGFBP3
	PTGER4
	PTGER2
	PTGER3
	ACHE
	KDM4E
	NTRK2

	CCNB3
	CCNB1
	CCNB2
	FLT1
	FLT4
	AURKC
	FEN1
	EDNRA
	AVPR2
	IDH1
	MAPT
	HDAC3
	HDAC6
	PTK2
	PLK1
	PTGS2
	ABCB1
	SLC22A12
	TLR9
	MAOA
	TERT
	PTGS1
	MCL1
	ODC1
	FLT3
	HSP90B1
	HSPA1A
	CXCR4
	GCGR

	GPR84
	ALPL
	MMP1
	CCR4
	CXCR2
	PDE10A
	MAPKAPK5
	ALK
	PRKAA1
	CTSL
	NOX4
	HDAC1
	GPR35
	CXCR1
	EGLN1
	ALOX15
	HSP90AB1
	KCNMA1
	STS
	TNNC1
	TNNT2
	TNNI3
	SNCA
	PRKCA
	PRKCB
	PRKCE
	HRH3
	GRK6
	IGFBP3

	PTGER4
	PTGER2
	PTGER3
	ACHE
	KDM4E
	NTRK2
	CCNB3
	CCNB1
	CCNB2
	FLT1
	FLT4
	AURKC
	FEN1
	EDNRA
	AVPR2
	IDH1
	MAPT
	HDAC3
	HDAC6
	PTK2
	PLK1
